# Methane Alleviates Lung Injury through the IL-10 Pathway by Increasing T Regulatory Cells in a Mouse Asthma Model

**DOI:** 10.1155/2022/6008376

**Published:** 2022-06-30

**Authors:** Ying Yao, Xiaoyong Miao, Liping Wang, Zhengyu Jiang, Lingxia Li, Ping Jiang, Yifei Wang, Aixia Jin, Na Li, Changli Wang, Kezhe Tan, Yan Meng, Jinjun Bian, Yan Zhang, Xiaoming Deng, Jianping Cao

**Affiliations:** ^1^Department of Anesthesiology, Navy Medical Center, Naval Military Medical University, Shanghai 200052, China; ^2^Department of Anesthesiology, Fuzhou General Hospital of PLA, Fuzhou, China; ^3^Department of Gastroenterology, Changhai Hospital, Naval Military Medical University, Shanghai, China; ^4^Department of Biochemistry and Psychopharmacology, Shanghai Mental Health Center, Shanghai Jiaotong University School of Medicine, Shanghai 200030, China; ^5^Shanghai Key Laboratory of Psychotic Disorders, Shanghai Mental Health Center, Shanghai Jiaotong University School of Medicine, Shanghai, China; ^6^Department of Anesthesiology and Intensive Care, Changhai Hospital, Second Military Medical University, Shanghai, China

## Abstract

Allergic asthma is associated with allergen-induced airway hyperresponsiveness and inflammatory cell infiltration. While moderate-to-severe asthma with refractory symptoms is difficult to treat, methane is protective against organ damage. In this study, an asthmatic mouse model was established. Airway resistance under acetylcholine stimulation in asthmatic mice and histology of lung tissue injury were determined. EOS infiltration was determined by flow cytometry. Enzyme-linked immunosorbent assays (ELISAs) were performed for the determination of relevant cytokine levels in asthmatic mice with or without methane treatment. The potential mechanisms of methane under anti-IL-10 antibody intraperitoneal intervention were assessed by ELISA and flow cytometry. Pulmonary T regulatory cells (Tregs) were analyzed by flow cytometry, and anti-CD25 antibody was used to block them. Immunoblot analysis was performed to evaluate if methane played a role in the asthmatic lungs via the NF-*κ*B and MAPKs pathways. The results showed that methane significantly improved airway compliance, relieved asthma-induced lung injury, and reduced EOS accumulation and inflammatory mediators in the lungs of ovalbumin-treated asthmatic mice. Anti-IL-10 treatment diminished the ameliorating effect of methane on asthma. In addition, methane enhanced pulmonary Tregs in asthma, which could be blocked by the anti-CD25 antibody. Further analysis revealed that methane decreased p-p65/p65 and p-p38/p38 expression. In conclusion, methane is a readily available and inexpensive molecule potentially suitable for human use, which can alleviate asthma-induced lung injury and EOS infiltration through the IL-10 pathway by increasing Tregs and decreasing NF-*κ*B and p38 MAPK in a mouse model.

## 1. Introduction

Asthma, or bronchial asthma, is essentially a chronic airway inflammation involving a variety of cells and cell components and is featured by airway hyperresponsiveness (AHR), resulting in recurrent and persistent wheezing, shortness of breath, and coughing. Uncontrolled inflammatory responses play a key role in asthma, a condition mainly characterized by excessive proliferation of T lymphocytes and infiltration of eosinophils (EOS), promoting alveolar injury and airway remodeling [1–3]. As indicated by the Global Asthma Report 2018, there are 339 million patients living with asthma, with approximately 20% of them having uncontrolled, moderate-to-severe disease with asthmatic symptoms in spite of standard and regular therapy such as inhaled corticosteroid (ICS) and *β*2 agonists [2]. Therefore, a new and specific therapy is required to handle such a dilemma.

Among various T lymphocyte subpopulations, T regulatory cells (Tregs) belong to one that plays a critical part in immune response, participating in the remission process of Alzheimer disease, inflammatory bowel diseases, asthma, etc. [4–6]. They maintain a balance of immune homeostasis and play a role in immune tolerance to allergens via a variety of mechanisms including cell-to-cell contact and TGF-*β* and/or IL-10 secretion [7, 8], thus attenuating inflammatory responses caused by eukocytes such as T effector cells and T cytotoxic cells (Tc). Depletion of Tregs reduced inflammation induced by effector T cells. It is known that Tregs express CD25 and have a high nucleic expression of FoxP3, which is a critical regulator for their development and differentiation [5]. A previous animal study indicated that CD4^+^CD25^+^FoxP3^+^ Tregs exerted an antiasthmatic effect in the asthma mouse model [3]. Another clinical research showed that Tregs induced by glucocorticoids expressed and generated more IL-10 to suppress the process of inflammation and thus alleviate lung injury, which explained the effectiveness of glucocorticoids in standard asthma therapy [9].

IL-10, as an anti-inflammatory mediator, is expressed by Tregs and plays a role in inhibiting immune response, thus protecting the body from inflammatory damage [10]. To be specific, IL-10 alleviates lung injury by inhibiting TNF-*α* production and neutrophil activation, which is essential in the early stage of inflammation [11]. In many animal models of infectious diseases, Treg-induced IL-10 expression decreases morbidity and mortality and facilitates tissue repair. In addition, IL-10 induced by Tregs is necessary in some autoimmune models [12]. The production of the anti-inflammatory cytokine (CK) IL-10 is involved in the Toll-like receptor (TLR) signaling, recruiting Myd88 inducing p38 MAPK cascade activation and indirect activating NF-*κ*B and ERK. The p38 MAPK and ERK can activate mitogen- and stress-activated kinases (MSK), which have two subsets MSK1 and MSK2, nuclear proteins capable of regulating IL-10 generation [13]. Allergy is related to allergen-specific IgE production, as well as the increase in allergen-specific T cell counts. Among these T cells, most of them are Th2 cells, which can produce characteristic CKs like IL-4, IL-5, and IL-13 to promote eosinophilic inflammatory response [14, 15].

Methane or methane saline (MS), a small molecule modulator, has been found to downregulate immune responses via antioxidative mechanisms [16–18]. Evidence has shown the correlation of methane generation in the gastrointestinal tract with constipation in irritable bowel syndrome, delaying and increasing the amplitude of ileal peristaltic contractions [19]. Moreover, exogenous methane application is correlated with redox modulation and mitochondrial dysfunction alleviation [20]. And through anti-inflammatory, antioxidant, and antiapoptotic effects, methane is reported to exert beneficial impacts on diabetic retinopathy, carbon-monoxide poisoning encephalopathy, autoimmune hepatitis, and one-time exhaustive exercise capacity caused by concanavalin A [14, 21–23]. In addition to being able to be distributed to all parts of tissue and organs, diffuse methane can freely cross cell membranes to activate intracellular pathways, such as inhibiting translocation of NF-*κ*B [24]. Studies have shown that methane limited the LPS-induced NF-*κ*B/MAPKs signaling in macrophages and reduced immune responses in mice through enhancing PI3K/AKT/GSK-3*β*-mediated IL-10 expression [24]. However, whether MS could help treat asthma remains to be elucidated.

Employing a mouse model, the motivation and novelty of the work is to clarify if methane exerts protective effects on asthma and if so, to identify underlying mechanisms. Asthma characterized by AHR was evaluated and the pulmonary pathology caused by immune cell infiltration was assessed with or without methane injection. In addition, the effect of methane on airway inflammatory cells, CKs, and IgE was evaluated. Possible mechanisms were explored by assessing IL-10, Tregs, CD25, NF-*κ*B, and MAPKs, as these components might be related to methane-induced immunosuppression. Our work supports the role of methane as a novel target for asthma treatment.

## 2. Material and Methods

### 2.1. Sources of Animals and Main Reagents

The study was conducted following institutional guidelines on ethics after obtaining approval from the Animal Care and Use Committee at Changhai Hospital, Second Military Medical University, Shanghai, China. Our experiments on animals took place in the Laboratory Animal Center of Second Military Medical University. BALB/c female mice (6–8-week-old, weighing 20 g) were bred in a temperature-controlled room under a nearly 12 : 12 h dark-light cycle and fed with a standard chow diet and water ad libitum. All minor surgeries in mice were performed under inhaled sevoflurane anesthesia at a low concentration of 1.5% *v*/*v* to reduce the suffering of mice. Chick ovalbumin (OVA) was supplied by Sigma (Cat No. A5503-1G, Thermo Scientific, United States) and from Imject Alum (Cat No. 77161, Thermo Scientific, Germany); aluminum hydroxide gel was obtained: solution A: 1 mg OVA dissolved in 1 ml NS; solution B: 50 *μ*l solution A + 50 *μ*l of aluminum hydroxide gel. Anti-IL-10 (clone JESS-16E3, Cat No. 16-7101), rat IgG 2b *κ* isotype control (clone eB149/10H5, Cat No. 16-4031-85), and anti-CD25 (clone PC61.5, Cat No. 14-0251-81) antibodies were all purchased from eBioscience Inc. (United States).

### 2.2. Methane-Rich Saline Preparation

Methane was freshly prepared the day before animal experiments to secure its constancy as follows. It was sealed for storage in a small biogas tank, followed by 3 h of high pressure (0.4 MPa) dissolution in normal saline (NS) till saturation, for the final storage (4°C) under atmospheric pressure. Methane determination in the NS was performed with a gas chromatography (GC-9860, Qiyang Co., Ltd., Shanghai, China) as reported by the published methods.

### 2.3. Asthma Induced by OVA in Mice

Initial sensitization of the animals was conducted via intraperitoneally injecting solution B on days 0, 7, and 14 of the study. Subsequently, nebulized 1% OVA in NS was atomized using an ultrasonic atomizer with an aerosol chamber (Yuwell Medical Equipment and Supply, Jiangsu, China), and the mixture was inhaled daily for half an hour from days 21 to 25. The described allergic asthma model included two processes, namely, intraperitoneal OVA sensitization and inhaling OVA, and thus, we used the term “OVA-primed and OVA-challenged” to describe the whole procedure. After 24 h, those mice were euthanized using dislocation of cervical vertebra and the lung samples were extracted.

### 2.4. Experimental Protocol

To begin with, 18 animals were equally grouped into three groups, including the normal group (marked as N), the asthmatic group (marked as asthma) treated as mentioned above, and the MS-treated group (marked as asthma-MS) intraperitoneally administered with 16 ml/kg MS 30 min before inhalation on days 21 through 25. Afterwards, another 36 mice (6 mice per group, 6 groups) were included for further experiments. The N group and the asthma group were described as above. The asthma-MS mice were intraperitoneally treated with NS (still marked as asthma-MS), anti-IL-10 (marked as asthma-MS-anti-IL-10), isotype control (marked as asthma-MS-ISO), and anti-CD25 (marked as asthma-MS-anti-CD25). The additional three groups of mice were pretreated with 1.5 mg/kg antibody of anti-IL-10, isotype control, and anti-CD25, respectively, 48 h prior to MS injection.

### 2.5. Airway Resistance Assessment

On the 21^st^ day, all the mice were sent to the Central Laboratory, Huashan Hospital (Fudan University, Shanghai, China) for AHR analysis. The evoking concentrations of acetylcholine [25] were set at the ascending doses of 3.125, 6.25, and 12.5 mg/ml. The AHR values were calculated, and the measurement plot was created.

### 2.6. Histological Analysis

For histological evaluation of pulmonary tissues, we fixed mouse left lung in 10% formalin solution, after which the fragments were dehydrated, cleared, and final paraffin-embedded. After tissue sectioning, the slices 8–10 *μ*m in thickness were treated with haemotoxylin and eosin (H&E) staining for pathological analysis. Peribronchial and perivascular inflammation degrees were evaluated with a subjective scale ranging from 0 to 4 by referring to a previous study [3]. Precisely, the extent of cell infiltration in the mouse lung could be scored using the Leica microsystem (Germany) according to the following criteria: 0, no cells can be found; 1, a small amount of cells; 2, a ring of one cell deep; 3, a ring of 2-4 cells deep; and 4, a ring of >4 cells deep.

### 2.7. Bronchoalveolar Lavage Fluid (BALF) and Lung Tissue Collection

On day 26, the animals were anaesthetized by inhaling 1.5% sevoflurane and their chests were carefully opened. Right principal bronchus intubation was performed to acquire BALF that was lavaged twice with 1 ml NS. The harvested BALF was immediately put on ice and centrifuged (350 × *g*, 4°C) for 5 min. Cell pellets were then resuspended in 200 *μ*l NS, 2 *μ*l of which (1%) was stained with trypan blue for cell counting, and the numbers of viable cells were calculated using a hemocytometer (WZR-BM2s, Ailin Medical Instrument Co. Ltd., China). The left lung was extracted, put on ice, and minced by syringe to single-cell suspension. Prior to flow cytometry, the cell suspension from one lung was washed by adding up NS to 50 ml and centrifuged at 350 × *g*, 4°C for 5 min, as the acquired pellets were resuspended in 1 ml and 2 *μ*l of which (0.2%) was stained with trypan blue for cell calculation by the hemocytometer.

### 2.8. Flow Cytometry for EOS and Treg Counts in BALF and the Lungs

All flow cytometry analyses were performed using BD FACS Calibur (United States). After centrifuging (350 × *g*, 4°C) BALF for 5 min, the cell mass was adjusted to 1 × 10^5^ cells/100 *μ*l for fluorescence staining. EOS were stained with FITC-conjugated anti-mouse-CCR3 (clone HL3; BD), APC-conjugated anti-mouse-CCR3 (clone J073E5; Biolegend) and PerCP-conjugated anti-mouse-CD170 (clone E50-2440; BD) on ice in the dark for 10 minutes and marked as CD11c^−^CCR3^+^CD170^+^ cells in flow cytometry analysis. To analyze Tregs in murine lung, Foxp3 staining procedures were performed following the manufacturer's recommendations (Cat No. 72-5775, eBioscience, United States). Briefly, cells were dyed with surface antigens, including FITC-conjugated anti-mouse-CD4 (clone RM4-5; BD) and PerCP-conjugated anti-mouse-CD25 (clone PC61; BD) on ice in the dark for 10 min. Then, the cell suspension was fixed, washed, and permeabilized following the manufacturer's manual. This was followed by the immersion of the treated cells in the anti-mouse/rat Foxp3 antibody (clone FJK-16S; eBioscience) for half an hour at room temperature in the dark. Tregs were marked as CD4^+^CD25^+^FOXP3^+^ cells. Flow cytometry data were analyzed by FlowJo 7.6 (United States).

### 2.9. CK Analysis by ELISA in BALF and Serum

First, we centrifuged the BALF at 350 × *g* and 4°C for 5 min to collect pellets as the cell component, and the supernatant isolated was treated with another 5 min of centrifugation (10000 × *g*, 4°C). The suspension was centrifuged twice, and the final supernatant was aliquoted to measure CK levels. As for serum samples, mice were anesthetized by inhaling sevoflurane and blood was sampled via cardiac puncture. Following collection of the whole blood, we left it at room temperature to clot by keeping it undisturbed for 15 to 30 min and then discarded the clot via 10 min of centrifugation (1000 − 2000 × *g*, 4°C). The supernatant obtained was a designated serum. We measured BALF and serum IgE and CK levels with sandwich ELISA kits (eBioscience, United States), strictly following the manufacturer's manuals. Samples were read at 450 nm using a BioTek Instruments (United States) in the 96-well plate.

### 2.10. SYBR Green Real-Time PCR

After separating total RNA from lung tissue by TRIzol lysis buffer (Takara, Japan), we measured its concentration, purity, and quality using the absorbance intensity at 260 nm, the ratio of 260 to 280 nm absorbance, and the absorbance ratio at 260/230 nm. The reverse transcription procedure was accomplished with the use of a PrimeScript RT Reagent Kit supplied by Takara. Measurements of IL-4, IL-5, and IL-10 concentrations were realized via the StepOne Plus Real-Time PCR System (Applied Biosystems, United States) by calculating their 2^Ct^ value deviation with *β*-actin. The primers used can be found in Table 1.

### 2.11. Western Blot

Mouse lung tissue was homogenized using protein lysis buffer (Thermo Fisher, United States) plus protease inhibitor cocktails (Sigma, United States). After 10 min of high-speed centrifugation (13000 × *g*, 4°C), protein concentration was measured using a BCA protein assay kit (Thermo Fisher, United States). Electrophoresis was accomplished in 10% SDS-PAGE gel (Life Technologies, United States), as each lane had the equivalent amounts of protein. Subsequently, gels were transferred to polyvinylidene difluoride (PVDF) membranes purchased from Thermo Fisher, United States. These membranes were then blocked in 5% nonfat dried milk for 1 h and immersed overnight at 4°C in the following first antibodies all provided by Cell Signaling Technology: p-p65 (1 : 1000), p65 (1 : 1000), p-ERK (1 : 2000), ERK (1 : 1000), p-JNK (1 : 2000), JNK (1 : 1000), p-p38 (1 : 1000), and p38 (1 : 1000). This was followed by 2 h of room temperature incubation with a HRP-conjugated secondary antibody (1 : 2000, Thermo Fisher, United States). Prior to each incubation, membrane rinsing with TBST was carried out three times for 5 min each. The visualization of protein bands was finally conducted via an ECL kit (Thermo Fisher, United States) under a UVP imager (United States).

### 2.12. Statistical Analysis

All analyses in this study were performed using Prism v.5.0. (GraphPad Software Inc., San Diego, CA, USA). Statistical significance of data represented by mean ± SEM was determined by Student's two-tailed *t*-test within two groups or ANOVA followed by Dunnett or Bonferroni's correction if there were multiple groups. Kruskal-Wallis one-way analysis was used for nonparametric analyses. *P* < 0.05 was the significance level. Specifically, ∗ and ∗∗ denote *P* < 0.05 and *P* < 0.01, respectively, versus the normal group; # and ## denote *P* < 0.05 and *P* < 0.01, respectively, versus the asthma group; △ and △△ denote *P* < 0.05 and *P* < 0.01, respectively, versus the asthma-MS group.

## 3. Results

### 3.1. MS Mitigates Acetylcholine-Induced AHR and Pathological Changes of OVA-Induced Lung Injury

**Figure 1:**
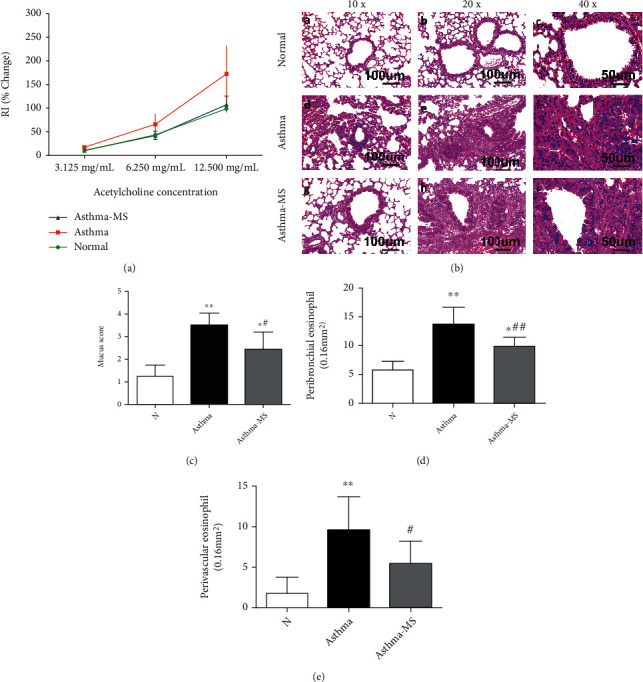
MS protects mice from OVA-induced lung injury as assessed by airway resistance and histology. Airway resistance measurements were performed by a FinePointe RC system supported by Huashan Hospital (Fudan University, Shanghai, China) (a); the histology shows 10x, 20x, and 40x magnification images of H&E staining of the lungs (b); the mucus score was assessed by cell infiltration: 0, no cells were observed; 1, a small amount of cells; 2, a ring of one cell deep; 3, a ring of 2-4 cells deep; and 4, a ring of more than four cells deep (c); eosinophil counts in peribronchial (d) and perivascular regions (e) of the lungs from three groups of mice were identified by the pink-stained cytoplasm. The data are presented in the form of the mean ± SEM (*n* = 6 per group). ∗ and ∗∗ denote *P* < 0.05 and *P* < 0.01, respectively, versus control group; # and ## denote *P* < 0.05 and *P* < 0.01, respectively, versus asthma group.

The nature and severity of asthma can be evaluated by detecting changes in pulmonary resistance and compliance triggered by acetylcholine inhalation-induced bronchoconstriction, which is the gold standard for the assessment and diagnosis of asthma [25]. We measured the airway resistance in normal mice, as well as OVA-primed and OVA-challenged animals treated with or without MS (Figure 1(a)). Airway resistance was found to be elevated in the asthmatic mice compared with the control mice, and MS treatment remarkably attenuated the increment of airway resistance (*P* < 0.05).

To evaluate the antiallergic and anti-inflammatory actions of MS against OVA-lung injury, we analyzed the HE-stained murine left lung tissue under the microscope. We found nearly no lung tissue inflammation in the control group (A, B, and C in Figure 1(b)), while exacerbated inflammation in the mouse lungs in the OVA-primed and OVA-challenged group, with EOS and other leukocyte infiltrations and more erythrocytes visible in many alveolar cavities (D, E, and F in Figure 1(b)). Remarkably, in the MS-treated groups, mice displayed a better lung pathological status compared with OVA-primed and OVA-challenged group mice without MS treatment (G, H, and I in Figure 1(b)). The inflammation scores were evidently lower in asthma-MS-treated mice versus asthmatic mice (Figure 1(c)). The lungs in the asthma-MS groups exhibited less extensive EOS infiltration surrounding the bronchi (Figure 1(d)) and blood vessels (Figure 1(e)) than the lungs in the asthma group. This demonstrates favorable effects of MS on AHR and pathological changes of asthma-induced lung injury.

### 3.2. MS Mitigates OVA-Induced Lung Injury by Reducing EOS Infiltration in BALF and Inhibiting Asthma-Related Inflammatory CKs in BALF, the Lungs, and Serum

**Figure 2:**
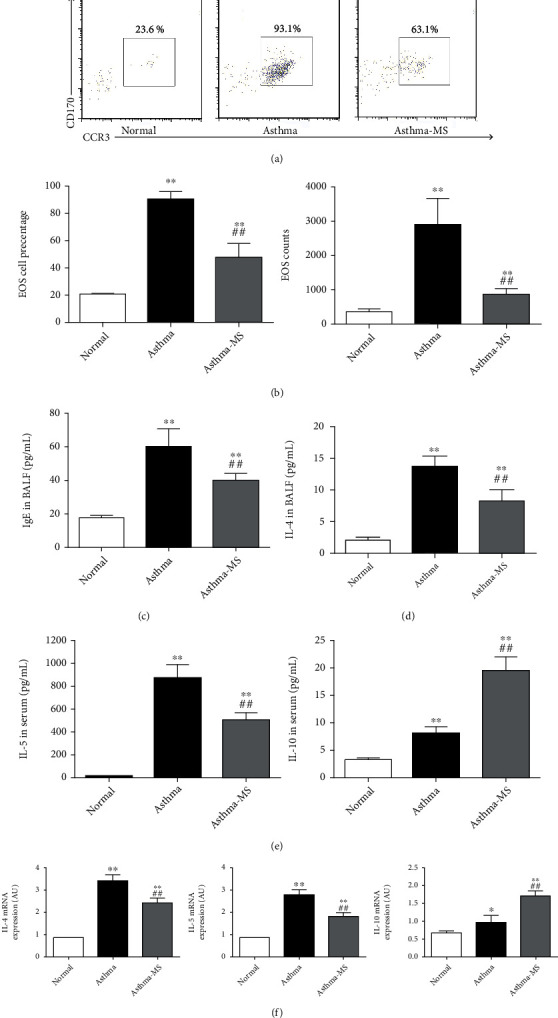
Methane protects mice from OVA-induced lung inflammation as assessed by EOS, IgE, and protein and mRNA expression of asthma-related cytokines. The representative flow cytometry plot indicates the EOS cell percentage in BALF (a) and these data were visualized (b). IBALF IgE (c) and IL-4 (d) levels, as well as serum IL-5 and IL-10 concentrations, were measured and analyzed by ELISA (e). In the transcriptional analysis, mRNA levels of IL-4, IL-5, and IL-10 in the lungs were evaluated and analyzed by quantitative reverse transcription PCR (f). The data are presented in the form of the mean ± SEM (*n* = 6 per group). ∗ and ∗∗ denote *P* < 0.05 and *P* < 0.01, respectively, versus control group; ## denote *P* < 0.01, respectively, versus asthma group.

To precisely analyze EOS infiltration in the lungs, we performed flow cytometry to measure the cell percentage and cell counts of EOS in both BALF and the lungs. The results showed an increase in the percentage of EOS cells treated with OVA treatment compared with the normal group, which was prevented by treatment with MS, as evidenced both in the BALF (Figures 2(a) and 2(b)). OVA treatment increased the total number of leukocytes, as well as EOS cell counts in BALF. MS injection ameliorated the increase of EOS counts caused by asthma both in the BALF (Figure 2(c)).

To determine the impacts of MS on IgE and CK levels in asthmatic mice, we measured BALF and serum concentrations of IgE, IL-4, IL-5, and IL-10 using ELISA and qRT-PCR. In significantly increased BALF, IgE and IL-4 levels were observed in OVA-primed and OVA-challenged mice compared with controls, and MS partially reversed this effect, suggesting that MS mitigated BALF IgE (Figure 2(d)) and IL-4 (Figure 2(e)) elevation caused by OVA. In the analysis of serum CKs, OVA-treated mice exhibited increased IL-5 and IL-10 secretion in serum, while MS treatment attenuated IL-5 elevation but enhanced IL-10 elevation (Figure 2(f)). Note that IL-10 is a potent anti-inflammatory factor. Through transcriptional analysis, it was found that the asthma group had upregulated IL-4, IL-5, and IL-10 expression in lung tissue than the normal group, while MS treatment augmented IL-10 and attenuated the elevation of IL-4 and IL-5. Collectively, the favorable antiasthmatic action of MS might be interpreted and even mediated by reducing EOS infiltration, upregulating IL-10 expression, and downregulating IgE, IL-4, and IL-5 concentrations.

### 3.3. Anti-IL-10 Intervention Attenuates the Protective Effect of MS

**Figure 3:**
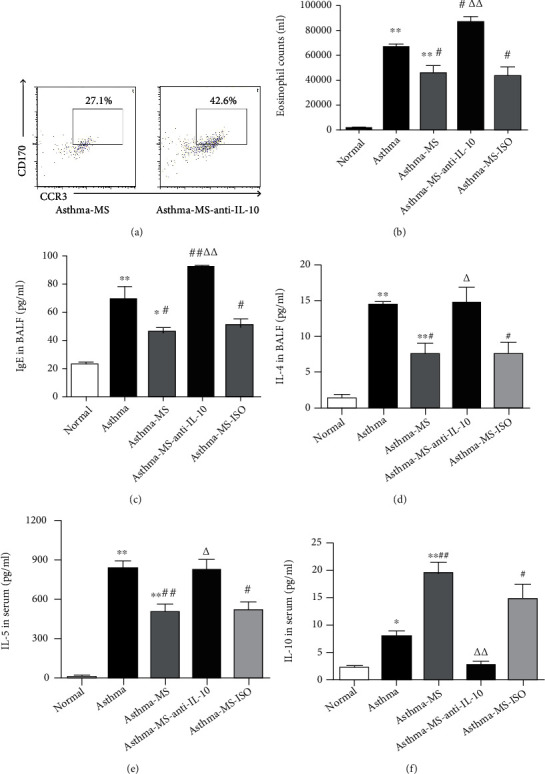
Anti-IL-10 partially reverses MS-induced pulmonary protection. The representative flow cytometry plot indicates the EOS cell percentage in one lung (a), and the data are graphed (b). The IgE and IL-4 levels in BALF were measured and analyzed by ELISA (c), and the IL-5 and IL-10 cytokine levels in serum were assessed and analyzed by ELISA (d). The data are presented in the form of the mean ± SEM (*n* = 6 per group). ∗ and ∗∗ denote *P* < 0.05 and *P* < 0.01, respectively, versus control group; # and ## denote *P* < 0.05 and *P* < 0.01, respectively, versus asthma group; △ and △△ denote *P* < 0.05 and *P* < 0.01, respectively, versus asthma-MS group.

IL-10, a multipotent CK that alleviates inflammation, is the production of several kinds of immune cells such as macrophages and T lymphocytes. IL-10 production plays a pivotal part in downregulating excessive inflammatory processes and preventing tissue injury triggered by cytotoxic immune responses [10]. Hence, we speculated that MS might mediate anti-inflammatory response via the IL-10 pathway. To clarify the antiasthmatic action of MS, we additionally administered anti-IL-10 antibodies to the asthma-MS mice (labeled as the asthma-MS-anti-IL-10 group), with the relevant isotype control as a control (labeled as the asthma-MS-ISO group). It was found that anti-IL-10 treatment significantly increased the EOS count compared with the MS-treated mice without anti-IL-10 medication (Figures 3(a) and 3(b)), and the isotype control injection did not alter the protective effect of MS (Figure 3(b)). Furthermore, we analyzed changes in IgE and CK resulting from anti-IL-10 treatment. Similarly, anti-IL-10 inhibition partially affected the amelioration effect of MS (Figures 3(c) and 3(d)). Anti-IL-10 medication eliminated the MS-induced decline of IgE and IL-4 in BALF (Figure 3(c)) and attenuated MS-induced inhibition of IL-5 and augmentation of IL-10 in serum (Figure 3(d)). To summarize, anti-IL-10 intervention attenuates the favorable role of MS in lung tissue.

### 3.4. MS Elevates Pulmonary Cell (CD4^+^ CD25^+^ Foxp3^+^) Counts Diminished by Anti-CD25 Antibody

**Figure 4:**
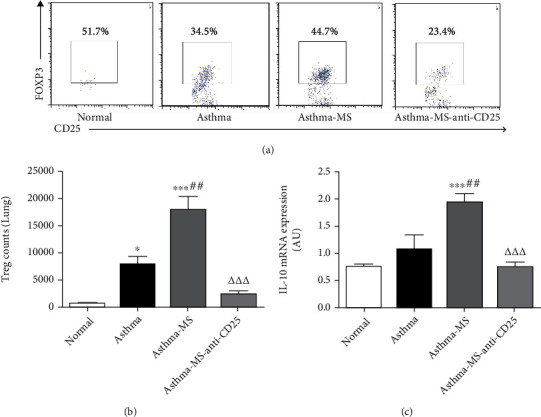
MS prevents excessive inflammatory response by stimulating Treg proliferation antagonized by anti-CD25 treatment. A representative flow cytometry plot indicates the Treg cell percentage in one lung (a) which is graphically presented in (b). IL-10 mRNA expression in the lungs by anti-CD25 antibody was analyzed (c). The data are presented in the form of the mean ± SEM (*n* = 6 per group). ∗ and ∗∗∗ denote *P* < 0.05 and *P* < 0.001, respectively, versus control group; ## denote *P* < 0.01, respectively, versus asthma group; △△△ denotes *P* < 0.001, respectively, versus asthma-MS group.

Previous studies have shown that elevation of CD4^+^ CD25^+^ Foxp3^+^ cells (Tregs) in lung tissues is beneficial to tissue injury induced by extensive inflammatory responses in the asthmatic mouse model [3]. Therefore, we investigated to see whether Tregs participated in the process of MS-induced protection by analyzing Treg counts in the lungs. Our results demonstrated that OVA sensitization and challenge increased the total Treg count in the lungs compared to the control group (Figure 4(b)). Surprisingly, MS was capable of raising Treg cell percentage and numbers and IL-10 gene expression in the lungs compared with the asthma group (Figures 4(a)–4(c)). However, anti-CD25 treatment had a significantly inhibitory effect on MS-induced Treg elevation and IL-10 upregulation (Figures 4(a)–4(c)). Therefore, the anti-inflammatory effect of MS on asthma-induced lung injury is associated with pulmonary Tregs, which can be blocked by anti-CD25 treatment.

### 3.5. MS Negatively Regulates Inflammatory Responses in Asthma via NF-*κ*B and p38 MAPK Axis

**Figure 5:**
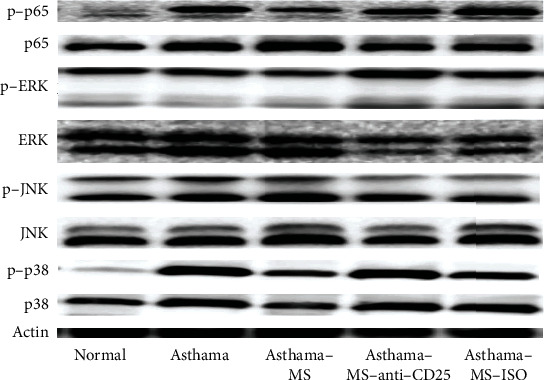
MS alleviates lung injury in asthmatic mice via the NF-*κ*B and MAPKs signaling pathways. Representative western blots of protein expression of p-p65, p65, p-ERK, ERK, p-JNK, JNK, p-p38, and p38.

Activation of the NF-*κ*B and MAPK pathways is known to play a key role in TLR-triggered proinflammatory CK production that leads to excessive inflammatory response and tissue damage [13, 24]. In addition, the anti-inflammatory CK IL-10 is regulated by MSK1 and MSK2, which are downstream nuclear proteins of NF-*κ*B, p38 MAPK, and ERK in the TLR signaling [13]. In this part, we explored the possible role played by MS in TLR-triggered phosphorylation of MAPKs and NF-*κ*B. The lung tissues of asthmatic mice had higher protein expression of p-p65/p65, p-JNK/JNK, and p-p38/p38 than the untreated mice (Figure 5). It seemed that MS had an inhibitory effect on p-p65/p65 and p-p38/p38 expression compared with the normal group, and anti-CD25 medication partially reversed the inhibition of MS. Consequently, MS might inhibit the NF-*κ*B and p38 signaling in the lungs by increasing Tregs.

## 4. Discussion

Our experimental results indicated that MS ameliorated asthma-induced AHR and decreased cellular infiltration in the lung epithelium, decreased EOS cell counts in BALF and the lungs, lowered proinflammatory CK (IgE, IL-4, and IL-5), and augmented anti-inflammatory CK (IL-10) expression. Anti-IL-10 antibody treatment can inhibit the favorable effect of MS. MS significantly elevated Treg counts and IL-10 expression, and reduced p-p65/p65 and p-p38/p38 protein expression in the asthmatic lungs, which were antagonized by anti-CD25 antibody treatment. To conclude, MS protects asthma-induced pulmonary insult through the IL-10, NF-*κ*B, and p38 MAPK pathway by increasing pulmonary Tregs.

Previous studies have shown that methane is a powerful small molecule capable of suppressing excessive inflammatory responses and protecting body tissues from damage [18, 24, 26]. Our study is the first to report that methane significantly reduced AHR in mice with asthma and protected the airway and the lungs from acute damage. The release of toxic mediators by EOS is one characteristic of allergic asthma [27]. Both our histological and cytometry analyses revealed that MS alleviated EOS and other immune cell infiltration into the alveolar spaces of asthmatic mice and relieved pulmonary mucus secretion. Anti-IgE is one therapeutic approach used for the treatment of asthma [28]. We measured IgE and relevant inflammatory CKs in both mRNA and secretive levels. The decline of IgE in BALF caused by MS leads us to consider that methane might be used for asthma therapy, particularly when ICS and *β*-agonist are ineffective.

T helper cells type 2 (Th2), another subset of T lymphocytes, is essential in the process of asthma, secreting CKs such as IL-4, IL-5, and IL-13 that directly impair lung tissue or indirectly facilitate the proliferation of IgE-generating B cells and EOS, contributing to AHR and airway remodeling [29]. IL-10 is reported to block the conversion from naïve CD4^+^ T cells to Th2, alleviating the symptoms of asthma. In addition, IL-10 is an anti-inflammatory factor normally released by M2c macrophages, which can prevent tissue damage from superfluous inflammation [30]. As methane dramatically alleviated AHR and protected lung tissues from excessive immune responses, we examined the relationship between MS and IL-10. Herein, the application of anti-IL-10 antibody confirms a hypothesis that MS might play its antiasthmatic role by activating the IL-10 pathway, potentially antagonizing the effect of Th2.

Tregs (CD4^+^ CD25^+^ FoxP3^+^) mainly act as an inhibitory cell to prevent inflammatory pathology and autoimmune diseases [10, 12, 31]. Some investigators consider Tregs as high IL-10-expressing cells and FoxP3 being vital for the differentiation, multiplication, and function of CD4^+^ CD25^+^ T cells [30]. Asthmatic patients have been confirmed to have a low level of FoxP3 in blood [31]. The present study indicated that MS significantly increased Tregs and IL-10 gene expression in the lung tissue, which could be inhibited by anti-CD25 administration. Nevertheless, the use of anti-IL-10 antibody did not change pulmonary Tregs (data not shown). Tregs can be classified as either natural Tregs (nTreg) or inducible Tregs (iTreg). nTregs are classical Tregs expressing FoxP3 and CD4^+^CD25^+^, while iTregs are considered as nonclassical Tregs normally not expressing FoxP3 [5, 32, 33]. One possible reason why the anti-IL-10 antibody intervention did not seem to have an effect on Tregs in our study is that it may have decreased Foxp3^−^iTregs (unmeasured), but not the Foxp3^+^nTregs as measured in this study. Another possible explanation is that TGF-*β*1 is more critical than IL-10 in the initiation and generation of Tregs, and IL-10 might be downstream of Treg formation [34]. The third reason is that IL-10, the downstream molecule of Tregs, follows FoxP3^+^ Treg induction and formation, so the anti-IL-10 antibody has little effect on pulmonary Tregs.

Earlier studies found that MS inhibited mouse proinflammatory reactions by upregulating the PI3K/AKT-mediated IL-10 and suppressing the NF-*κ*B/MAPKs pathway in macrophages [24]. In addition, MSK1 and MSK2 are downstream nuclear proteins of NF-*κ*B and p38 MAPK, which are critical to IL-10 production [13]. Therefore, we analyzed p-p65/p65, p-ERK/ERK, p-JNK/JNK, and p-p38/p38 expression in the lungs. However, this study still has room for improvement. First, although we found that MS had the potential to treat asthma by inhibiting the NF-*κ*B and p38 MAPK signaling, which was partially reversed by anti-CD25 antibody treatment, more mechanisms remain to be elucidated. In future research, we can investigate whether methane affected the NF-*κ*B/MAPKs signaling in Th2 or EOS or B cells. Moreover, it is not clear whether MS can induce Treg formation in vitro and whether MSK1 and MSK2 in asthma are methane mediated.

## 5. Conclusion

Our findings demonstrate that MS injection can alleviate AHR, improve airway remodeling and lung pathology, decrease EOS count, and balance CK expression and secretion by immune cells in a mouse model of allergic asthma. Specifically, MS plays its antiallergic role through the activation of the IL-10 pathway via CD4^+^ CD25^+^ Foxp3^+^ augmentation and p38 MAPK and NF-*κ*B attenuation, antagonized by the anti-CD25 antibody. We will further analyze whether MS could induce Treg formation in vitro and whether MSK1 and MSK2 are mediated by methane in asthma. The study demonstrates the potential of MS as a new therapeutic target for the treatment of asthma.

## Figures and Tables

**Table 1:** Primer sequences.

Objects	Primer sequences (5′-3′)
IL-4	Forward (F): CCCCAGCTAGTTGTCATCCTG
	Reverse (R): CAAGTGATTTTTGTCGCATCCG
IL-5	F: TCAGGGGCTAGACATACTGAAG
	R: CCAAGGAACTCTTGCAGGTAAT
IL-10	F: GCTGGACAACATACTGCTAACC
	R: ATTTCCGATAAGGCTTGGCAA
*β*-Actin	F: AGCGGTTCCGATGCCCT
	R: AGAGGTCTTTACGGATGTCAACG

## Data Availability

The simulation experiment data used to support the findings of this study are available from the corresponding author upon request.
